# HIV-1 Buds Predominantly at the Plasma Membrane of Primary Human Macrophages

**DOI:** 10.1371/journal.ppat.0030036

**Published:** 2007-03-23

**Authors:** Sonja Welsch, Oliver T Keppler, Anja Habermann, Ina Allespach, Jacomine Krijnse-Locker, Hans-Georg Kräusslich

**Affiliations:** 1 Department of Virology, University of Heidelberg, Heidelberg, Germany; 2 European Molecular Biology Laboratory, Cell Biology and Biophysics Program, Heidelberg, Germany; Northwestern University, United States of America

## Abstract

HIV-1 assembly and release are believed to occur at the plasma membrane in most host cells with the exception of primary macrophages, for which exclusive budding at late endosomes has been reported. Here, we applied a novel ultrastructural approach to assess HIV-1 budding in primary macrophages in an immunomarker-independent manner. Infected macrophages were fed with BSA-gold and stained with the membrane-impermeant dye ruthenium red to identify endosomes and the plasma membrane, respectively. Virus-filled vacuolar structures with a seemingly intracellular localization displayed intense staining with ruthenium red, but lacked endocytosed BSA-gold, defining them as plasma membrane. Moreover, HIV budding profiles were virtually excluded from gold-filled endosomes while frequently being detected on ruthenium red–positive membranes. The composition of cellular marker proteins incorporated into HIV-1 supported a plasma membrane–derived origin of the viral envelope. Thus, contrary to current opinion, the plasma membrane is the primary site of HIV-1 budding also in infected macrophages.

## Author Summary

Macrophages are one of the major target cells for HIV-1 infection and play an important role in viral pathogenesis. Previous studies indicated that the pathway of HIV-1 particle morphogenesis is distinct in primary human macrophages, and this has been suggested to play a role in viral persistence. Early reports indicated that HIV-1 accumulates within apparently intracellular vacuolar structures, which were later identified as being of late endosomal origin. Endosomes were therefore suggested to comprise the budding and storage compartment for HIV-1 in primary human macrophages, from which infectious virus can be released in a regulated manner. In the present study, we show that HIV-1 budding occurs predominantly at the plasma membrane also in primary human macrophages. Using electron microscopy, we observed that the cell surface of macrophages displays an unexpectedly complex morphology with many protrusions and deep invaginations. HIV-1 budding occurs primarily at these invaginations that are clearly connected to the cell surface and do not belong to the endocytic compartment. Mature virus particles can remain trapped within such invaginations giving the appearance of an intracellular budding compartment. These results suggest a general pathway of HIV-1 morphogenesis with the plasma membrane as viral budding site.

## Introduction

HIV-1 is an enveloped retrovirus that acquires its envelope by budding through limiting membranes. CD4^+^ T cells and macrophages are the primary targets of HIV-1 and are commonly used to study virus replication in tissue culture. Infected macrophages constitute a long-lived reservoir for HIV persistence and rebound and thus pose a major challenge for HIV clearance from infected individuals (reviewed in [1–3]). Recently, differences in the site of virus assembly and budding have emerged as a major distinguishing feature of HIV-1 infection in macrophages and have been discussed as an important factor in virus persistence and dissemination.

In HIV-1-infected primary CD4^+^ T cells and most cell lines, assembly and budding occur at the plasma membrane [4–8], possibly involving lipid rafts [6,9]. In contrast, early ultrastructural studies implicated intracellular organelles as sites of HIV-1 budding in infected macrophages, such as the Golgi complex and vacuoles [10–13]. More recent immuno-electron microscopy (EM) studies of HIV-1-infected primary human macrophages provided strong support for the cell type–specific intracellular budding and proposed late endosomes (LE) and multivesicular bodies (MVB) as budding compartments in these cells [14,15]. These authors reported HIV-1 budding into large intracellular vacuoles bearing markers of LE/MVB, including tetraspanins (CD63, CD81, and CD82), lamp-1, and MHC-II. Accordingly, extracellular infectious virions were shown to carry tetraspanins, while being largely devoid of most GPI-anchored and cell adhesion proteins tested [14–17].

Topologically, HIV budding and the formation of intralumenal vesicles of the MVB are similar (away from the cytoplasm) and share the requirement for ubiquitin conjugation and recruitment of the cellular ESCRT-I and ESCRT-III complexes [18]. Thus, budding of HIV-1 at LE/MVB may be ideally suited to exploit the cellular pathway of exosome formation. These considerations, together with the described EM studies, led to the current view that HIV-1 assembles and buds almost exclusively from late endosomal membranes in macrophages [14,17]. The resulting intracellular accumulation of HIV-1 was proposed to be important for pathogenesis and dissemination since HIV-1 can be retained in an infectious state for prolonged periods of time inside macrophages [19] and may be released in a delayed manner similar to secretion of exosomes [20,21].

Contrary to the prevailing view of ESCRT-localization, our recent immuno-EM analysis of uninfected and HIV-1-infected primary human T cells and macrophages showed that the analyzed ESCRT-proteins (HRS, TSG101, AIP1/ALIX, and VPS4) localized to both the endosomal compartment and the plasma membrane [22]. No significant relocalization of ESCRT-proteins was observed in infected cells, even when a high producer T-cell line was analyzed [22]. Our frequent observation of plasma membrane budding in HIV-1-infected primary macrophages prompted us to re-assess HIV-1 budding site localization in macrophages. To this end, we applied a novel ultrastructural approach, which is independent of immunomarker distribution, to distinguish endosomal structures from the plasma membrane. Our results reveal that the plasma membrane is the predominant site of HIV-1 budding also in primary macrophages and suggest a general pathway of HIV-1 morphogenesis.

## Results

### EM Analysis of HIV-1-Infected Primary Human Macrophages Reveals Viral Budding at Vacuolar Structures and at the Plasma Membrane

We revisited HIV-1 assembly and budding in primary human monocyte-derived macrophages (MDM), which had been used in previous studies to demonstrate HIV-1 accumulation within intracellular compartments [10–15]. MDM cultures infected with either of the two macrophage-tropic HIV-1 strains Ba-L or YU-2 showed largely similar replication kinetics (unpublished data). Cells were fixed for EM at the peak of virus release, usually around 12–17 d after infection, and either embedded in Epon before sectioning or processed for cryo-sections followed by immunolabeling.

**Figure 1:**
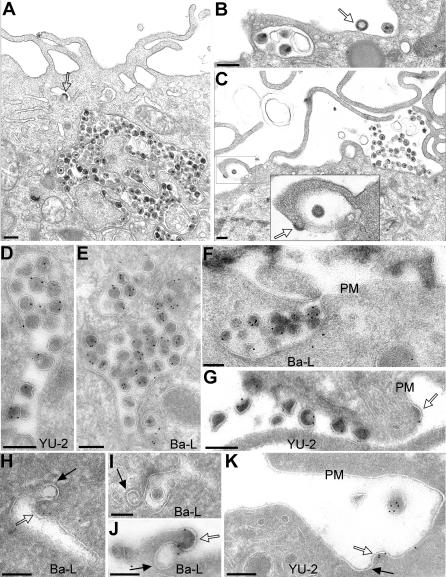
A Gallery of Primary Human MDM Infected with HIV-1 Strains YU-2 and Ba-L Cells were infected with HIV-1 strain Ba-L (A–C, E, F, and H–J) or YU-2 (D, G, and K). (A–C) Shows plastic-embedded sections displaying extra- and seemingly intracellular virions and budding profiles (open arrows). (D–K) Thawed cryo-sections were labeled with anti-capsid followed by 10 nm (D–G, J, and K) or 5 nm (H and I) protein A gold. (D and E) Labeled virions accumulated inside vacuolar structures. (F) Labeled virions in a plasma membrane invagination that is directly connected to the extracellular space. (G) A virus-budding profile (open arrow) and several virus particles on the plasma membrane. (H) A virus-budding profile (open arrow) next to a structure resembling a clathrin-coated pit (arrow) inside a vacuolar structure. (I) A virus-containing vacuole with a structure resembling a clathrin-coated pit (arrow). (J and K) Virus-budding profiles (open arrows) next to structures resembling clathrin-coated pits (arrows) on the cell surface. PM, plasma membrane. Bar, 200 nm.

Accumulation of mature HIV-1 particles with typical cone-shaped capsid morphology in large, seemingly intracellular vacuolar structures was readily detected in HIV-1 Ba-L- (Figure 1A and 1B) or YU-2- (not shown) infected cells. While virus-filled vacuolar structures were often observed, detection of typical HIV-1 budding structures was much less common. Importantly, early (Figure 1A and 1C) and late (Figure 1B) budding profiles were detected on the plasma membrane (Figure 1B and 1C; open arrows), as well as on membranes that appeared to be intracellular on thin sections (Figure 1A; open arrow). This result was independent of the method of monocyte enrichment or cultivation of MDM (unpublished data). Furthermore, infected and uninfected MDM revealed a complex surface architecture with numerous protrusions and cross-sectioned invaginations suggesting that some apparently intracellular structures may be connected to the cell surface.

Immuno-EM using antiserum against the HIV-1 capsid protein readily identified HIV-1 particles in large vacuolar structures with an apparent intracellular localization, both for strain YU-2 (Figure 1D) and Ba-L (Figure 1E and 1F). The limiting membrane of these structures was sometimes connected to the extracellular space, indicating that they represented deep plasma membrane invaginations (Figure 1F). Furthermore, virus particles were frequently detected in the extracellular space (Figure 1G). Many virus-filled vacuolar structures contained membrane protrusions similar to the cell surface (see below), and their limiting membrane sometimes displayed structures resembling clathrin-coated pits (Figure 1H and 1I). HIV-1 budding profiles were detected on the limiting membrane of vacuolar structures (Figure 1H) and on the plasma membrane (Figure 1G, 1J, and 1K). Budding profiles were often next to structures resembling clathrin-coated pits (Figure 1H, 1J, and 1K, arrows), even when budding occurred into seemingly intracellular vacuoles. The observation of significant plasma membrane budding and of hallmarks of the plasma membrane on the limiting membranes of seemingly intracellular vacuoles prompted us to characterize the origin of these vacuolar structures in more detail. Sections of YU-2- and Ba-L- infected MDM were morphologically indistinguishable, and we therefore used Ba-L-infected MDM throughout the remainder of the study.

### An Immunomarker-Independent Approach to Discriminate Plasma Membrane from Endocytic Compartments

**Figure 2:**
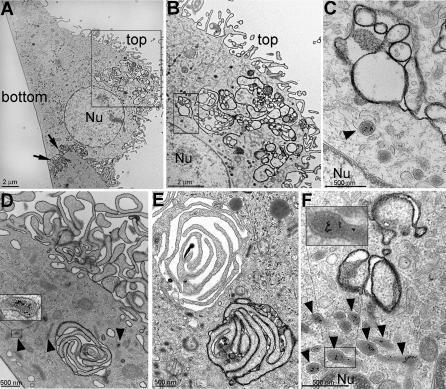
RR Staining of Uninfected MDM Reveals a Complex Plasma Membrane Organization MDM were fed with BSA-gold and fixed in the presence of RR. (A) A cell profile at low magnification contacting a neighboring cell. The smooth left side was previously attached to the substrate (indicated as “bottom”); the right side displaying many RR-positive protrusions is the side facing the extracellular medium (“top”). Seemingly intracellular structures also appear RR-positive. At the tight-fitting contact zones between two cells (arrows) the stain appears more electron-dense than on single lipid bilayers. (B) Higher magnification of the boxed area in (A) showing the collection of vacuolar-like invaginations stained with RR. (C) Higher magnification of the boxed area in (B) showing a RR-positive structure next to a BSA-gold-filled endosome (arrowhead). (D) Comparison of RR-positive cell surface protrusions and similar RR-positive protrusions, which appeared intracellular in this section. Arrowheads indicate gold-filled endosomes. The inset in (D) shows an enlargement of a BSA-gold-filled endosome in the boxed area. (E) Two identical looking structures with stacked protrusions; in the upper one the membranes are not RR-stained, indicating that not all vacuolar structures are accessible to the stain. (F) A collection of BSA-gold-filled endosomes (arrowheads) next to RR-stained vacuolar structures to emphasize the differences in size and morphology. The inset in (F) shows an enlargement of a BSA-gold-filled endosome in the boxed area. Nu, nucleus. Bars, 2 μm (A and B) and 500 nm (C–F).

Previous studies of HIV-1 morphogenesis in infected cells relied on immunolabeling of cellular proteins to identify membrane compartments. To allow an immunomarker-independent identification of the plasma membrane and endosomes, we combined two established EM methods in a novel ultrastructural approach. This involved labeling of endocytic compartments by internalizing BSA-gold followed by staining of the plasma membrane with ruthenium red (RR) during fixation. RR is a membrane-impermeant dye, which binds to carbohydrate moieties on the cell surface [23,24]. Because of its small size, RR can readily penetrate into membrane invaginations [25], while fixation at 4 °C prevents its internalization. Upon post-fixation, RR forms an electron-dense precipitate detectable on Epon-sections, and this method has been used to study HIV-1 entry in macrophages [26]. Our previous experiments had shown that over 75% of all endosomal structures of primary human macrophages, including LE, were filled with BSA-gold after 2 h of internalization [22]. Accordingly, the combination of BSA-gold uptake and RR staining should unequivocally identify membrane structures as being of endosomal or plasma membrane origin. This was first tested on uninfected MDM to validate our approach (Figure 2).

Figure 2A shows MDM morphology at low magnification; the smooth cell surface facing to the left was previously attached to the culture dish and is therefore poorly stained with RR. The complex upper surface is facing to the right, with many finger-like surface protrusions clearly stained with RR (Figure 2B). Two neighboring cells form a tight-fitting contact zone with interdigitating membranes (Figure 2A, arrows). Importantly, the limiting membranes of large, seemingly intracellular vacuolar structures that were heterogeneous in size and shape were also stained with RR, thus defining them as plasma membrane–derived (Figure 2A–2F). These structures generally had no apparent connection to the cell surface in the plane of the section and were often found deep inside the cell (Figure 2B and 2C). No RR labeling was observed on the nuclear membrane and on membranes of the endoplasmic reticulum, Golgi complex, or mitochondria. RR-positive vacuoles never contained BSA-gold and BSA-gold-labeled endosomes were always devoid of RR (Figure 2C–2F), confirming the specificity of our approach. BSA-gold-labeled endosomes were generally more electron-dense, often round or oval-shaped, and significantly smaller than the RR-stained vacuoles.

The RR-positive structures often enclosed stacked membranes resembling plasma membrane protrusions (Figure 2D and 2E). Morphologically identical RR-negative structures were also observed, sometimes next to RR-positive structures (Figure 2E). They were never filled with BSA-gold (e.g., Figure 2E) and are thus unlikely to be of endocytic origin. Their failure to stain with RR suggested that they are not directly connected to the cell surface, while their morphology strongly suggested a plasma membrane origin. Lack of staining may be explained by inaccessibility to the stain, however, as supported by inefficient staining of the cell surface attached to the substrate (Figure 2A).

### HIV-1 Buds from the Plasma Membrane and Accumulates in Plasma Membrane–Derived Structures in MDM

**Figure 3:**
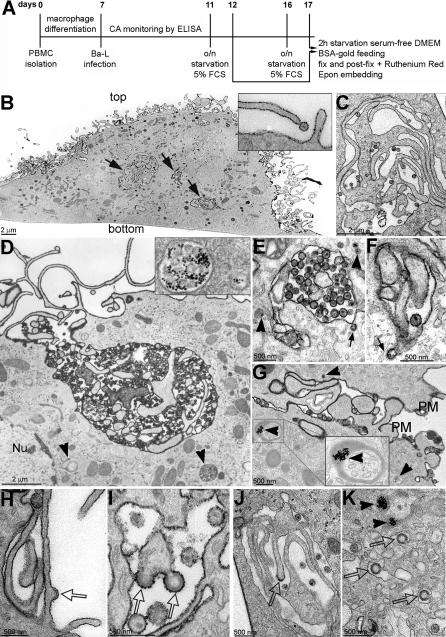
HIV-1 Buds and Accumulates in RR-Stained Domains (A) Flow diagram of macrophage differentiation, infection, and processing for EM. CA, capsid protein. (B) Low magnification profile of an infected MDM displaying RR stain on the cell surface and on seemingly intracellular plasma membrane invaginations (arrows). Note that the bottom surface (indicated as “bottom”) that was previously attached to the substrate is not RR-stained, whereas the top surface is. The inset shows a mature virus particle on the tip of a finger-like plasma membrane protrusion. (C) Collection of RR-stained protrusions with RR-stained mature virus particles. (D) Large cell-surface invagination that is directly connected to the RR-stained cell surface and contains numerous RR-stained virions. The inset shows a multivesicular BSA-gold-filled endosome inside the same cell. (E and F) RR-stained vacuole-like structures harboring virus particles and displaying structures resembling clathrin-coated pits (small arrows). Arrowheads in (E) indicate gold-filled endosomes that are not RR-stained and significantly smaller than the virus-filled structures. (G) RR-stained virus particles accumulating between the RR-stained plasma membranes of two neighboring cells; arrowheads, BSA-gold-filled endosomes. The inset in (G) shows an enlargement of a BSA-gold-filled endosome in the boxed area. (H–K) Virus-budding profiles (open arrows) (H) on the RR-stained cell surface and (I) on a RR-stained cell surface invagination. (J) Early virus bud on the tip of a finger-like protrusion in a vacuolar structure devoid of RR and BSA-gold. (K) Late virus buds on RR-negative membranes next to BSA-gold-filled endosomes (arrowheads). Nu, nucleus; PM, plasma membrane. Bars, 2 μm (B–D) and 500 nm (E–K).

Next, this approach was used to identify the membranes of HIV-1-containing compartments and of viral budding sites in MDM. Cells infected with HIV-1 Ba-L were fed with BSA-gold followed by RR staining at the peak of virus production (Figure 3A). Infected cells displayed a complex surface organization similar to uninfected MDM with many seemingly intracellular vacuoles (Figure 3B, arrows) containing mature HIV-1 with cone-shaped cores. Importantly, the limiting membrane of many of these vacuoles and the surface of the enclosed virions were strongly RR-positive, similar to the cell surface and extracellular virions (Figure 3B–3I). Identical virus-filled structures were observed in cells that had not been starved and filled with BSA-gold prior to fixation, clearly showing that this phenotype was not affected by the pre-treatment (unpublished data). The RR-positive virus-harboring structures contained protrusions reminiscent of the plasma membrane (Figure 3C), sometimes displayed structures resembling clathrin-coated pits (Figure 3E and 3F), and were never labeled with BSA-gold, also indicating that they were cell surface–derived. Figure 3D shows a large RR-positive vacuolar structure with many protrusions and numerous mature HIV-1 particles that is directly connected to the cell surface. RR-positive early and late HIV-1 budding profiles were observed at the cell surface (Figure 3H) and on vacuolar structures (Figure 3I), confirming that virus assembly and release did occur on plasma membrane invaginations that appeared intracellular in thin sections.

Similar to the observations with uninfected MDM, a subset of virus-harboring vacuolar structures was not stained with RR, while the morphology of these structures was indistinguishable from the corresponding RR-positive compartment (Figure 3J and 3K). HIV-1 budding sites were also observed on such RR-negative membranes (Figure 3J and 3K). These structures were always devoid of BSA-gold and thus did not belong to the endocytic pathway. Gold-filled endosomes were often detected in the vicinity of HIV-1-containing vacuoles (Figure 3D, 3E, and 3K) and appeared significantly smaller than virus-filled vacuoles. This was quantified by measuring the surface area of virus-filled vacuoles and endosomes (Table 1). The vacuolar structures covered 1–3.1 μm^2^, while endosomes had an average size of 0.08 μm^2^ and were thus ten to 40 times smaller.

**Table 1:**
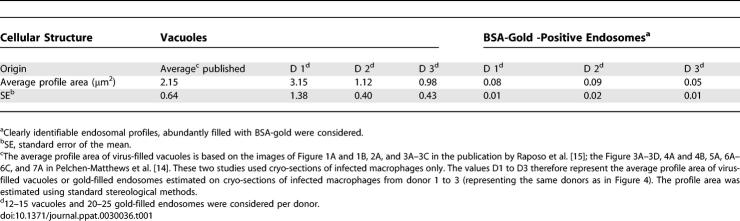
Average Profile Area of Virus-Filled Vacuoles and Gold-Filled Endosomes in Primary Human Macrophages

**Figure 4:**
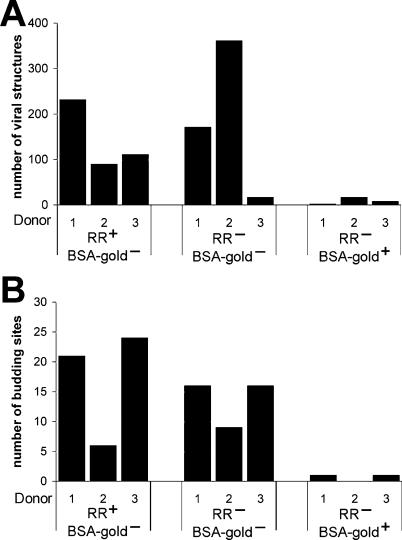
Quantification of Membrane Structures Where HIV-1 Accumulates and Buds Three types of cellular membranes, either surrounding free virus or displaying budding sites, were considered: (i) RR-positive without internalized BSA-gold (RR^+^, BSA-gold^−^), (ii) RR-negative without BSA-gold (RR^−^, BSA-gold^−^), and (iii) RR-negative with BSA-gold (RR^−^, BSA-gold^+^). No RR-positive, BSA-gold-positive structures were observed. (A) Numbers of viral structures (sum of free virus particles and budding profiles) seen on the three types of membrane structures in infected MDM prepared from three blood donors (designated 1–3). Free virus included mature virions with a typical conical core and immature particles displaying an electron dense Gag shell. Budding sites include “late buds” consisting of spherical profiles that are connected to a membrane via a stalk (see, for instance, Figure 3K and “early buds”) and half-moon-shaped membrane profiles with an electron-dense Gag shell on their concave side (see, for instance, Figure 3H). (B) Distribution of budding profiles over the same three membrane structures and of the same three donors as in (A).

For a quantitative assessment of virus-specific structures, their localization in infected MDM from three different donors was attributed to one of three compartments depending on the presence or absence of RR and/or BSA-gold. Over 94% of HIV-1-specific structures were detected in compartments devoid of internalized gold. These structures were mostly stained with RR in the case of two donors (60% and 80% of all structures, respectively; Figure 4A, donors 1 and 3), while the majority of virus accumulated in RR-negative (and BSA-gold-negative) structures in the case of donor 2. Accumulation of HIV-1 in a certain compartment does not necessarily imply that budding occurs from this compartment, and the localization of viral budding sites was therefore also quantified. The absolute number of budding profiles was relatively small since 91%–97% of all HIV-1-specific structures observed corresponded to free virus particles. Importantly, quantification of budding profiles revealed that 98%–100% localized to BSA-gold-negative structures with the relative distribution between RR-positive (40%–60%) and RR-negative budding profiles being similar to that observed for free viruses (Figure 4B).

### Distribution of Cellular Marker Proteins Supports a Plasma Membrane Origin of the Viral Envelope

Although the RR-negative virus-containing structures were morphologically indistinguishable from the RR-positive ones and never contained BSA-gold, they could have represented a population of endosomes that is not part of the active endocytic pathway and therefore inaccessible to the endocytic tracer. The tetraspanins CD63, CD81, and CD82 and the lysosomal membrane protein lamp-1 were previously shown to localize to endosomal compartments and to be incorporated into the viral envelope of macrophage-derived HIV-1 particles [14,27–29]. Since the presence of these membrane proteins in the HIV-1 envelope has been taken as important evidence that HIV-1 buds into endosomes in MDM [14], we tested their localization in infected MDM and included CD44 as a plasma membrane marker [30].

**Figure 5:**
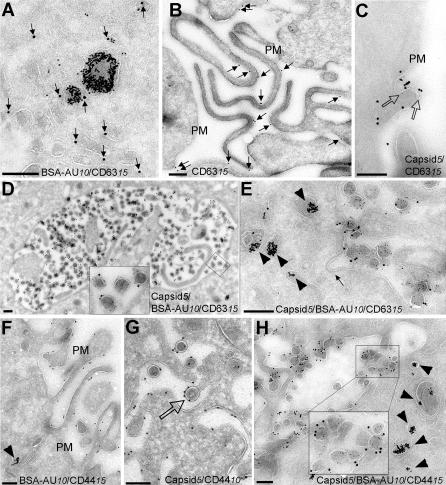
Localization of CD63 and CD44 in HIV-1-Infected MDM HIV-1 strain Ba-L-infected MDM were fed with 10 nm BSA-gold (except in G) for 2 h before fixing. Thawed cryo-sections were single-labeled with anti-CD63 (A and B, 15 nm gold) or anti-CD44 (F, 15 nm gold); or double-labeled with anti-capsid (C–E, G, and H, 5 nm gold) and anti-CD63 (C–E, 15 nm gold); or anti-CD44 (G, 10 nm gold and H, 15 nm gold). (A) CD63 (arrows) on endosomes efficiently filled with BSA-gold and on tubular vesicular membranes nearby. (B) CD63 (arrows) on plasma membrane protrusions of two adjacent cells. (C) A virus-budding profile labeled for anti-capsid (5 nm gold, open arrows) and anti-CD63 (15 nm gold) at the plasma membrane. (D and E) Virions inside vacuolar structures, labeled with anti-capsid (5 nm), are significantly labeled with anti-CD63. The inset in (D) shows the enlargement of double-labeled virions in the boxed area of (D). CD63 also localizes to BSA-gold-filled endosomes (arrowheads in E). Note that the virus-filled structure in (E) is devoid of 10 nm BSA-gold and displays a structure that resembles a clathrin-coated pit (small arrow). (F) CD44 at the plasma membrane. (G and H) CD44 on virions and on a budding profile (open arrow in G). The inset in (H) is an enlargement of the boxed area in (H) and shows several double-labeled virions inside a vacuolar structure. Note that CD44 is excluded from BSA-gold-filled endosomes (arrowheads in F and H). PM, plasma membrane. Bars, 200 nm.

As expected, CD63 was predominantly found on membranes of BSA-gold-filled endosomes (Figure 5A). Significant labeling for this marker was also detected on the plasma membrane (Figure 5B) as reported previously [17,31]. CD63 labeling at the plasma membrane was uneven with areas of higher and lower density (unpublished data). The tetraspanins CD81 and CD82 and the lysosomal protein lamp-1 exhibited a similar labeling pattern as CD63, albeit with a lower labeling efficiency (Figure S1).

Consistent with the cell surface localization of this marker, CD63-positive budding profiles were observed at the plasma membrane (Figure 5C). Extracellular virions (unpublished data), particles inside vacuolar structures, and the limiting membranes of these structures were positive for CD63 (Figure 5D and 5E), and, to a lesser extent, lamp-1, CD81, and CD82 (Figure S1) as previously reported [14]. For each of these markers, labeling of the limiting membrane was similar on all virus-containing structures, suggesting that they do not segregate into separate classes. Importantly, CD44, which localized almost exclusively to the plasma membrane and was absent from BSA-gold-filled endosomes (Figure 5F–5H), was also readily detected at budding sites (Figure 5G) and efficiently incorporated into HIV-1 particles (Figure 5F and 5H).

**Figure 6:**
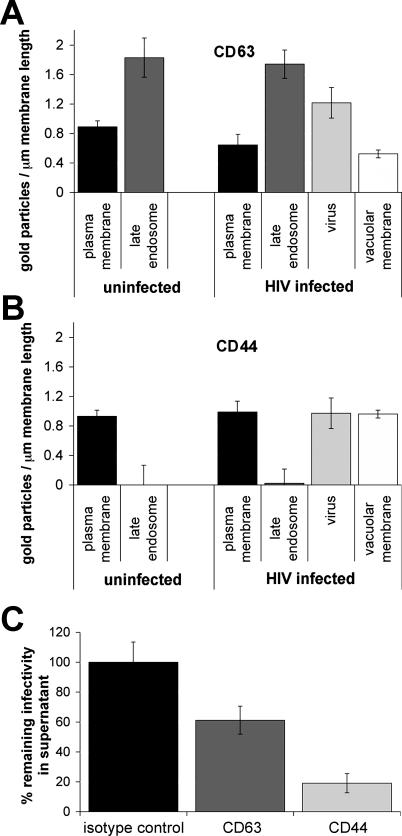
Quantification of Labeling Density and Virus Precipitation by Antibodies to Cellular Membrane Proteins (A and B) Labeling densities for CD63 and CD44 were quantified on late endosomes (identified by the presence of internalized gold and by their morphology) and the cell surface (only clearly identifiable plasma membrane was considered) of uninfected or infected MDM. In infected cells, the labeling densities on viral particles (“virus”) and on the limiting membrane of virus-containing vacuolar structures (“vacuolar membrane”) was also included. Values represent the average from two independent labeling experiments and two different grids per experiment. The quantification was performed for MDM from the same donor 1 as in Figure 4; the results for the two other donors are shown in Figure S2. Error bars denote standard error. (C) Remaining infectivity in supernatants of HIV-infected MDM after virus immunoprecipitation with anti-CD63 or anti-CD44, analyzed by infection of a HIV-1-permissive reporter cell line. Values obtained after precipitation with isotype control antibodies were set at 100%. Virus was obtained from MDM culture supernatant of the same donor as in (A and B). Error bars denote standard deviation.

Quantitative analysis of the labeling densities of CD63 (Figures 6A and S2) and CD44 (Figure 6B) revealed that they were virtually identical on the plasma membrane and on the limiting membrane of virus-filled vacuolar structures, supporting a cell surface origin of the latter. The labeling density of CD63 in uninfected and infected MDM was 2- to 3-fold higher on endosomes than at the plasma membrane, and the labeling density on virions was intermediate between the two (Figure 6A). In contrast, CD44 was almost exclusively found at the plasma membrane and virus-filled vacuolar membranes and was excluded from gold-filled endosomes (Figure 6B). The CD44 labeling density was identical on the plasma membrane, the limiting membranes of virus-filled vacuolar structures, and the membranes of enclosed HIV-1 particles (Figure 6B), supporting the plasma membrane origin of the particle membrane.

To correlate the immuno-EM data with the membrane composition of infectious HIV-1 particles derived from MDM, we immunoprecipitated MDM-derived virus with monoclonal antibodies to CD63, CD44, or control antibodies, respectively, as previously described [14,32]. Precipitation with anti-CD63 reduced infectivity by ∼40% (Figure 6C), and this was similar for virus derived from MDM of different donors (Figure S2). These data are consistent with previous studies showing that CD63 is incorporated into infectious HIV-1 from MDM [14,16]. An even stronger titer reduction was observed when virus was precipitated with antibodies against the plasma membrane marker CD44 (∼80%, Figures 6C and S2). Collectively, these results support the conclusion of predominant plasma membrane budding of HIV-1 in primary human macrophages.

## Discussion

The current view of HIV-1 morphogenesis distinguishes two fundamentally different pathways of virus release. This implies differences in targeting and membrane interaction of viral components, depending on the host cell. HIV-1 budding is readily detected by EM at the plasma membrane of T-lymphocytes, which are morphologically small and characterized by a large nucleus and a small cytoplasmic compartment. In infected macrophages, on the other hand, HIV-1 has long been reported to accumulate within large vacuoles [10–13], recently described as being of endosomal origin [14,15,21]. This assignment was based on immunolocalization of cellular proteins in combination with the intuitively appealing concept of common use of the ESCRT-machinery for MVB formation and HIV release at the same site. Here, we report that the seemingly intracellular virus-filled vacuoles are largely continuous with the plasma membrane and conclude that HIV-1 budding occurs predominantly at the plasma membrane also in macrophages. This is mainly based on the following observations: (i) the frequent detection of HIV-1 budding sites at the plasma membrane, (ii) the predominant HIV-1 budding and accumulation in RR-positive structures, (iii) the virtual exclusion of budding sites and HIV-1 particles from BSA-gold-filled endosomes, (iv) the size of virus-filled compartments being much larger than that of endosomes, and (v) the composition and labeling density of cellular proteins in the viral envelope supporting its plasma membrane origin. It is important to note that the sizes of virus-filled structures in this and previous studies were very similar (Table 1; [14,15]), implying that these compartments are the same. Thus, our experimental findings applying previously used techniques are entirely consistent with prior reports, while our interpretation—largely based on an immunomarker-independent approach—is fundamentally different.

The incorporation of specific cellular membrane proteins into the HIV-1 envelope has been taken as evidence for the origin of the viral membrane [32,33]. This interpretation suffers from the fact that HIV-1 produced from T cells—where the virus buds through the plasma membrane—carries the same marker proteins (reviewed in [34]). The distribution of cellular proteins is generally not exclusive to a single membrane system, however, and CD63 and other tetraspanins, while clearly enriched at LE, are also detected at the plasma membrane [17,35]. We found a 2- to 3-fold difference in CD63 density when comparing the endosomal and plasma membrane, with an uneven distribution of CD63 in different regions of the latter. This observation is consistent with two recent studies reporting the presence of tetraspanin-enriched microdomains in the plasma membrane of T cells and cell lines, from which HIV-1 was suggested to bud preferentially [31,36]. We observed a higher labeling density for CD63 in the HIV-1 membrane compared to the plasma membrane or membranes of virus-filled structures, consistent with HIV-1 budding from CD63-rich plasma membrane microdomains also in MDM. Plasma membrane budding was supported by the efficient labeling of HIV-1 buds and particles with anti-CD44, which localized almost exclusively to the plasma membrane. Furthermore, infectious virus was almost quantitatively precipitated by anti-CD44. This had also been observed in previous studies, where anti-CD44 precipitated >95% of infectious HIV-1 [16,37]. Thus, the collective studies on incorporation of cellular membrane proteins into HIV-1 support a plasma membrane origin of the viral envelope, independent of the infected host cell.

To define the budding membrane of HIV-1 in infected macrophages in an immunomarker-independent manner, we combined two established EM methods in a novel ultrastructural approach. Endosomes were functionally labeled with BSA-gold and the plasma membrane was stained with the membrane-impermeant dye RR [23,24,38]. Importantly, RR staining and BSA-gold never colocalized to the same compartment and no RR staining of internal organelles was detected, validating our approach. HIV-1 commonly accumulated in large, seemingly intracellular RR-positive structures, which were thus defined as plasma membrane invaginations. Although budding structures were much more rare, they were also observed at the RR-stained limiting membrane of such seemingly intracellular invaginations (and not on gold-filled endosomes), and the enclosed virus was stained with RR as well. The RR-positive compartment contained the majority of HIV-1 particles and budding sites, but they were also observed at RR-negative structures. We suggest that these virus-filled structures were similar, if not identical, to RR-stained invaginations and were also derived from the plasma membrane, but have not been accessible to RR for technical reasons. RR staining was poor for the substrate-adherent plasma membrane of MDM, and this would thus be expected for structures connecting to this surface as well. Notably, the size and morphology of RR-positive and RR-negative structures was identical, the latter also displayed occasional structures resembling clathrin-coated pits and membrane protrusions, and virus-filled structures were consistently CD44-positive. Furthermore, the density of both CD63 and CD44 was identical for plasma membrane and virus-filled vacuolar structures as would be expected if they represent the same compartment. Finally, labeling of the limiting membrane with antibodies against tetraspanins (CD63, CD81, and CD82) was consistent for all virus-containing structures, indicating that these structures comprise a homogenous population.

A direct connection of the virus-filled compartment to the cell surface within the plane of the section was only detected in rare cases, which may not be surprising given the complex three-dimensional architecture of the MDM plasma membrane. Images of virus-filled structures in direct membrane continuity with the cell surface have also been published previously [15], but were interpreted as exocytic vacuoles releasing virus budded at endosomal membranes. We consider this interpretation unlikely because virus-filled structures were devoid of BSA-gold, displayed features of the plasma membrane (see above), and were CD44-positive. Moreover, the large number of RR-positive structures would require a major part of the endomembrane system to be involved in exocytosis at any time, which cannot be reconciled with the main function of macrophages as phagocytic scavengers.

Our conclusion of plasma membrane budding of HIV-1 in primary human macrophages is in complete agreement with a report published while this work was under review [39]. These authors based their conclusion on kinetic analyses of Gag trafficking and virus release in the presence of various inhibitors and on targeting mutants of HIV-1 Gag and suggested that the intracellular pool of HIV-1 in macrophages may be due to phagocytosis of virions after their initial release at the plasma membrane [39]. Our ultrastructural analysis of primary human macrophages incubated overnight with large amounts of HIV-1 revealed virtually no viral particles in the endosomal compartment, however, arguing against this interpretation (unpublished data). The experiments presented here showed that uninfected and HIV-1-infected macrophages acquire a complex plasma membrane organization with many protrusions and tightly interdigitated cell contact zones as well as deep invaginations that on sections resemble large intracellular vacuoles. These structures enlarge the surface of the plasma membrane and may be physiologically relevant to sense the environment and to engulf particles to be destroyed. It is tempting to speculate that these highly convoluted parts of the plasma membrane may be different in nature compared to the cell surface that directly faces the growth medium. HIV-1 buds at this complex surface membrane, and infectious virions may be trapped in membrane pockets and accumulate there. Since infected macrophages are known to persist for months, these infectious virions could be released over extended periods of time, consistent with a recent report of prolonged storage of infectious HIV-1 in MDM [19]. Although this scenario is similar to the delayed exosome-release hypothesis [20], it is likely that the regulation of virus storage and release underlie an entirely different mechanism. Most importantly, however, our study suggests a universal morphogenesis pathway for HIV-1 with virus release occurring predominantly at the plasma membrane, independent of the host cell.

## Materials and Methods

### Reagents and antibodies.

All tissue culture reagents were from GIBCO BRL (http://www.invitrogen.com), unless otherwise indicated. EM chemicals were from EMS (http://www.emsdiasum.com) and Uranyl acetate was from Fluka (http://www.sigmaaldrich.com). All Epon embedding solutions, propylene oxide, and RR were from Serva (http://www.serva.de). Bovine Albumine Fraction V (Biomol, http://www.biomol.com) coupled to 10 nm gold was prepared as described [40]. Anti-CD63 mAb 1B5 (used for immuno-EM) was a kind gift from M. Marsh, anti-CD63 mAb FC5.01 was from Zymed (Invitrogen, http://www.invitrogen.com). Anti-lamp-1 mAb H4A3 was from DSHB (http://www.uiowa.edu/∼dshbwww/info.html), anti-CD44 mAb F10-44-2 from Chemicon (http://www.chemicon.com), and rabbit-anti-mouse IgG from ICN Biomedicals (http://www.mpbio.com). Anti-CD81 mAB M38 and anti-CD82 mAB M104 were kind gifts from F. Berditchevski. Anti-nPKCθ IgG2a (E-7, purchased from Santa Cruz Biotechnology, http://www.scbt.com) was used as isotype control. Polyclonal rabbit antiserum against the HIV-1 capsid protein was raised against bacterially expressed and purified protein.

### Cells, viruses, and infections.

Cultures of primary human MDM were routinely prepared from Ficoll gradient-purified peripheral blood mononuclear cells isolated from single, healthy, HIV-1-seronegative blood donors (DRK Blutspendezentrale, Mannheim, Germany) by adherence and were differentiated in culture for 6–8 d as described [22], in the presence or absence of recombinant human M-CSF (5 ng/mL; R & D Systems, http://www.rndsystems.com). Alternatively, MDM were differentiated from CD14 magnetic bead-selected monocytes from peripheral blood mononuclear cells according to the manufacturer's protocol (Miltenyi Biotec, http://www.miltenyibiotec.com). Details regarding MDM cultivation, infection with the macrophage-tropic HIV-1 strains YU-2 [41] or Ba-L (purchased from Advanced Biotechnologies, http://www.abionline.com), BSA-gold feeding, and RR staining are provided in Figure 3A.

### BSA-gold feeding and RR stain.

Primary human MDM were either left untreated or starved overnight in DMEM with 5% fetal calf serum (DMEM/5%), followed by an additional 2-h starvation in serum-free DMEM prior to BSA-gold feeding. Cells were either washed three times with ice-cold 20 mM EDTA/PBS and fixed directly or incubated for 2 h with BSA-gold (final OD_520_ = 10) in DMEM/10% containing 10% human AB-positive serum at 37 °C, conditions that have been shown to fill at least 75% of all endocytic compartments in MDM including late endosomes and lysosomes [22,29]. BSA-gold-filled cells were placed on ice and washed as above to remove excess BSA-gold and to partially detach the cells from the culture dish prior to fixation. All cells were fixed with 2.5% glutaraldehyde in 0.1 M ice-cold Na-Cacodylate buffer (pH 7.2) containing 0.5 mg/ml RR for 1 h, during which time cells were allowed to warm up to room temperature. Cells were then washed with 0.1 M Na-Cacodylate buffer (pH 7.2), post-fixed with 2% OsO_4_ in the same buffer containing 0.5 mg/ml RR for 1 h at room temperature before routine embedding in Epon as described [42].

### Immuno-EM.

Cryo-sections of fixed cells were prepared as described [43] and immunolabeled as before [42,44]. Sections were examined with a Zeiss EM10 (http://www.zeiss.com). The quantification of CD63 and CD44 labeling density was carried out and calculated essentially as described [22,42]. Values represent counts from at least two different grids and two independent labeling experiments per cell donor.

### HIV-1 immunoprecipitation assay.

Immunoprecipitation of HIV-1 from culture medium of HIV-1 Ba-L-infected MDM was essentially done as described [14,32] using anti-CD63 (FC5.01, Zymed), anti-CD44 (F10-44-2, Chemicon), or an IgG2a isotype control antibody (all at 5 μg/ml). After precipitation, the supernatants were analyzed for remaining unprecipitated infectious virus in a standard reporter cell assay as described [45].

## Supporting Information

Figure S1 Localization of Lamp-1 and Tetraspanins CD81 and CD82 and Incorporation into Virus Particles at Low Levels in HIV-1-Infected MDM Thawed cryo-sections of HIV-1 strain Ba-L-infected MDM, fed with 10 nm BSA-gold for 2 h prior to fixation, were either labeled with anti-CD81 (B and F) or CD82 (A, C, and G) and 15 nm gold (A–C, F, and G) or double-labeled with anti-lamp-1 (15 nm gold) and anti-capsid (5 nm gold, D and E).
(A) CD82 (arrows) localizes to BSA-gold-filled endosomes (arrowheads) at low levels.
(B and C) CD81 and CD82 (arrows) both localize to the plasma membrane.
(D and E) Lamp-1 (arrows) localizes to BSA-gold-filled endosomes (arrowheads in E) and is incorporated at low amounts into virus particles accumulating in vacuolar structures.
(F and G) Incorporation of CD81 (arrows in F) and CD82 (arrows in G) at low levels into virus particles that accumulate inside vacuolar structures. The arrowhead in (G) points at a BSA-gold-filled endosome.
Note that the vacuolar structures in (D–G) are devoid of 10 nm BSA-gold. Bars, 200 nm.
(15 MB TIF)

Figure S2 Labeling Densities of CD63 and Virus Precipitation with Anti-CD63 and Anti-CD44 in HIV-1-Infected MDM from Different Donors (A–C) Labeling densities of CD63 over different membranes as defined in Figure 6, including MDM from two additional donors 2 and 3 (corresponding to the same donors 2 and 3 as in Figure 4). The labeling density for CD63 on the plasma membrane and limiting vacuolar membrane are similar (if not identical) for all three donors, whereas the labeling density on LEs is significantly higher. Values represent the average from two independent labeling experiments and two different grids per experiment. Error bars denote standard error.
(D) Remaining infectivity in supernatants of HIV-infected MDM after virus immunoprecipitation with anti-CD63 or anti-CD44 as described in Figure 6C. Results are shown for MDM from donors 1 and 3 (corresponding to those named equally in Figures 4, 6, and A–C of Figure S2), for a third independent donor (donor 80), and commercially available HIV-1, strain Ba-L, derived from a multi-donor pool of MDM. Each virus was immunoprecipitated in triplicate. Error bars denote standard deviation.
(1.5 MB TIF)

### Accession Numbers

The accession numbers for the entities discussed in this study are from the Swiss-Prot database (http://www.expasy.org/sprot). They include Swiss-Prot entry name CD44_HUMAN, protein name CD44 antigen, gene name *CD44, LHR* (P16070); Swiss-Prot entry name CD63_HUMAN, protein name CD63 antigen, gene name *CD63, MLA1, TSPAN30* (P08962); Swiss-Prot entry name CD81_HUMAN, protein name CD81 antigen, gene name *CD81, TAPA1, TSPAN28* (P60033); Swiss-Prot entry name CD82_HUMAN, protein name CD82 antigen, gene name *CD82, KAI1, SAR2, TSPAN27* (P27701); and Swiss-Prot entry name LAMP1_HUMAN, protein name lysosome-associated membrane glycoprotein 1, gene name *LAMP1* (P11279).

## Abbreviations

EM: electron microscopy
LE: late endosome
MDM: monocyte-derived macrophage
MVB: multivesicular body
RR: ruthenium red
